# Ethnoracial disparities in cognition are associated with multiple socioeconomic status-stress pathways

**DOI:** 10.1186/s41235-021-00329-7

**Published:** 2021-10-09

**Authors:** Sarah K. Letang, Shayne S.-H. Lin, Patricia A. Parmelee, Ian M. McDonough

**Affiliations:** 1 Department of Psychology, The University of Alabama, 505 Hackberry Lane, BOX 870348, Tuscaloosa, AL 35487 USA; 2 Alabama Research Institute on Aging, Tuscaloosa, USA

**Keywords:** Cognition, Weathering hypothesis, Perceived stress, Socioeconomic status, Health disparities

## Abstract

Systemic racism can have broad impacts on health in ethnoracial minorities. One way is by suppressing socioeconomic status (SES) levels through barriers to achieve higher income, wealth, and educational attainment. Additionally, the weathering hypothesis proposes that the various stressful adversities faced by ethnoracial minorities lead to greater wear and tear on the body, known as allostatic load. In the present study, we extend these ideas to cognitive health in a tri-ethnic sample of young adults—when cognition and brain health is arguably at their peak. Specifically, we tested competing mediation models that might shed light on how two key factors caused by systemic racism—SES and perceived stress—intersect to explain ethnoracial disparities in cognition. We found evidence for partial mediation via a pathway from SES to stress on episodic memory, working memory capacity, and executive function in Black Americans relative to non-Hispanic White Americans. Additionally, we found that stress partially mediated the ethnoracial disparities in working memory updating for lower SES Black and Hispanic Americans relative to non-Hispanic White Americans, showing that higher SES can sometimes reduce the negative effects stress has on these disparities in some cognitive domains. Overall, these findings suggest that multiple pathways exist in which lower SES creates a stressful environment to impact ethnoracial disparities cognition. These pathways differ depending on the specific ethnoracial category and cognitive domain. The present results may offer insight into strategies to help mitigate the late-life risk for neurocognitive disorders in ethnoracial minorities.

## Significance statement

Systemic racism has broad effects on mental and physical health. However, it can also negatively impact cognition in ethnoracial minorities such as Black and Hispanic Americans. These cognitive disparities are important to understand because they give rise to negative and inaccurate group stereotypes of biological or innate differences that have plagued minorities for hundreds of years. These cognitive disparities also might be key causal factors that give rise to a greater prevalence and incidence rate of neurocognitive disorders in late life, including Alzheimer’s disease and vascular dementia. Although previous work has demonstrated that systemic racism impacts one’s socioeconomic level through decreased access to education, job attainment, and wealth and can impact the amount of stress one experiences, less work has shown how these factors intersect and how such intersections affect different types of cognition. This research reveals that socioeconomic status and stress can affect Black and Hispanic Americans differently. For example, some of the differences in cognition between Black and White Americans can be explained by the fact that lower socioeconomic status causes a great deal of chronic stress, which has long-term impacts on cognition. The same level of socioeconomic status was not associated with the same stress-related declines in cognition among Hispanic Americans. This research also reveals that systemic racism can have different impacts on the type of cognitive domain. These cognitive domain effects may explain why Black Americans are more likely to have mixed dementia (Alzheimer’s disease and vascular dementia) than other groups.

## Introduction

The daily discriminatory actions caused by systemic racism sustain disadvantages and disparities for ethnoracial minorities across different aspects of society (Johnson, 2020), including the work industry (Offermann et al., 2014), healthcare system (Williams et al., 2019), and criminal justice system (Hetey & Eberhardt, 2018). We consider “ethnoracial minorities” as Americans historically marginalized by the majority (non-Hispanic White) due to their race or ethnicity. According to the 2020 US Census (https://data.census.gov), 60% of Americans report being non-Hispanic White, with the largest proportion of other categories reported as 12% African-American/Black and 12% as Hispanic White. Although research on ethnoracial disparities in health has amplified over the past decade (Kim et al., 2010), less research has explored how certain systemic racism variables (e.g., increased levels of stress, lower educational opportunities, lower income) influence young adults’ cognition (Bair & Steele, 2010). Studying this gap may help researchers better understand how racism-related experiences often faced by ethnoracial minorities early in adulthood impact long-term or later life cognitive impairments often found relative to non-Hispanic White Americans. Furthermore, exploring such cognitive disparities related to systemic racism in young adulthood may shed light on developing preventative interventions for cognitive impairments later in life.

### Stress, socioeconomic status, and cognition

Several theories provide the foundation for understanding how stress differentially influences health between different ethnoracial categories. The minority stress model (Meyer, 2003) posits that minority groups experience unique and elevated rates of mental and physical health stressors often stemming from harassment, maltreatment, and discrimination. Internalized racism, defined as racist stigmas internalized following repeated experiences with discrimination, mediates the relationship between frequency of racist experiences and stress in Black Americans (Graham et al., 2016). Across all ethnoracial categories, allostatic load theory proposes that continued stress has a negative impact on one’s health through wear and tear on one’s body (Fisher & Reason, 1988; McEwen, 1998; McEwen & Stellar, 1993; Sterling et al., 1988). The weathering hypothesis combines these ideas and suggests that continued exposure to stress due to social and economic disadvantages results in greater allostatic load and health disparities for ethnoracial minorities, including an acceleration of biological aging (Forde et al., 2019; Geronimus, 1992).

### Stress

The hypothalamic–pituitary–adrenal (HPA) axis plays a critical role in the body’s reaction to stress and explains the mechanisms underlying allostatic load theory. When a stressful situation occurs, an individual’s HPA axis manages the stress by releasing a series of hormones, including corticotropin-releasing hormones, adrenocorticotropic hormones, and glucocorticoid hormones such as cortisol (Marin et al., 2011; Russell & Lightman, 2019). In the short term, such responses are adaptive and enhance cognitive resources to attend, encode, and react to threats in the environment by increasing blood flow to the muscles and brain. However, people are not meant to sustain elevated levels of these hormones for extended periods. If faced with chronic stressors, repeated, or prolonged activation of the HPA axis can result in excessive release of glucocorticoids to certain regions of the brain, including the hippocampus and prefrontal cortex (Harrell et al., 2011; Sandi, 2013). Notably, these two brain regions are known to be associated with multiple domains of cognition including memory and executive function (e.g., Oei et al., 2006). This excessive release increases the risk of hormonal dysregulation, such as glucocorticoid resistance, impairment in glucocorticoid receptor functioning, and brain atrophy, which in turn may have observable negative impacts on physical health, cognition, emotion regulation, well-being, and even life expectancy (Charles, 2010; Panter-Brick & Worthman, 1999; Russell & Lightman, 2019).

Although many objective events (e.g., a traffic jam) might be described as stressful, one’s bodily reaction to stress relies on an individual’s judgment or perception of that event as a stressor (Lazarus & Folkman, 1984; Oumohand et al., 2020). For this reason, one of the most popular means of measuring stress is through subjective reports of perceived stress, such as the Perceived Stress Scale (PSS; Cohen et al., 1983). Such measures often ask about multiple perceptions of stress over several weeks and have been interpreted as measures of chronic stress because they do not only pertain to single, acute events.

Among all ethnoracial categories, much research has investigated the effects of perceived stress on cognition in middle-aged to older adults. This research suggests that higher levels of stress are associated with poorer performance in several cognitive domains, including processing speed (Caswell et al., 2003; Korten et al., 2017), working memory (Oei et al., 2006; Oumohand et al., 2020; Schoofs et al., 2008), episodic memory (Gagnon et al., 2019; Zaheed et al., 2020), and executive functioning (Korten et al., 2017). The brain regions underlying these cognitive domains overlap with those most impacted by chronic stress (i.e., hippocampus and prefrontal cortex; Oei et al., 2006). Among ethnoracial minorities, stress has been argued to lead to a more rapid episodic memory decline in Black Americans than non-Hispanic White Americans (Zuelsdorff et al., 2020). Barnes et al. (2012) found that older Black Americans who reported experiencing higher levels of stress from systemic racism (i.e., perceived discrimination) showed worse performance not only in episodic memory but also in perceptual speed and global cognition. In a study of younger adults, experiencing stress from racism was found to trigger a depletion of one’s executive functioning–specifically in self-control (Bair & Steele, 2010). Lastly, when studying the differences among Hispanic Americans, Schmader and Johns (2003) found stress (i.e., stereotype threat) reduced working memory capacity relative to non-Hispanic White Americans.

### Socioeconomic status

Socioeconomic status (SES) also has been proposed as an important mediator of health (Adler et al., 1994; Gallo et al., 2005; Lynch & Kaplan, 2000). Income level and educational attainment are two common components of SES. Lower SES environments are associated with differences in neurocognitive development and potentially limit children from developing their cognitive potential, especially in the domains of language, working memory, and executive function (Farah et al., 2006; Merz et al., 2019). Accordingly, SES has been associated with altered brain structure and function mostly in the prefrontal cortex but also in the temporal lobe including the hippocampus (Merz et al., 2019), consistent with the cognitive correlates of SES. Moreover, SES can intersect with minority status to further suppress achievement on tests during childhood (Burchinal et al., 2011; Lubienski, 2002; Magnuson & Duncan, 2006).

Both income level and education often are lower among ethnoracial minority groups as a whole, including Black and Hispanic Americans compared to non-Hispanic White Americans (Burchinal et al., 2011; Kerckhoff & Campbell, 1977; Portes & Wilson, 1976). Other research suggests that income and education can mediate ethnoracial disparities in cognition, especially in late life (Lyketsos et al., 1999; Sachs-Ericsson & Blazer, 2005; Zahodne et al., 2017). Lastly, lower SES contributes to poor health generally, in part because of exposure to more frequent stress (Cundiff et al., 2020; Merz et al., 2019). Childhood SES, for example, has been associated with dysregulation of the HPA axis via altered cortisol levels (Merz et al., 2019).

### Present study

Although cognitive disparities among ethnoracial minority groups are fairly well-established, research investigating the impact of intersecting factors of systemic racism on these disparities in young adults is sparse. During young adulthood, the brain is at a point of maturation when cognitive performance across many domains is at its peak (Hartshorne & Germine, 2015). Young adulthood also is a critical period of identity formation and developing one’s place in the workforce. Stress and SES may impact this crucial period both immediately and over one’s life course. Recently, the literature regarding the impact of stress and SES on health disparities has been criticized for not critically testing the intersection of these factors to better understand specific pathways in which each operate (Cundiff et al., 2020). Our primary aim was to explore how stress and SES intersect to impact ethnoracial differences in cognition among Black and Hispanic Americans relative to non-Hispanic White Americans in young adulthood. Systemic racism and discrimination often experienced by ethnoracial minorities have been proposed as critical adversities that lead to allostatic load and premature weathering of health (Brody et al., 2014; Forde et al., 2019), potentially impacting one’s cognition. To explore this perspective, we tested three different manners in which stress and SES might influence ethnoracial differences in several cognitive domains, including episodic memory, working memory, and executive functioning.

First, we used parallel mediation to investigate whether perceived stress and SES independently contribute to ethnoracial differences in cognition. Second, we used sequential mediation to examine whether ethnoracial minorities with lower SES might be more prone to increased levels of stress, which in turn might be associated with lower cognition. Lastly, we used moderated mediation to investigate whether the relationship between stress and cognition in ethnoracial minorities might be specific to lower SES minorities, perhaps because higher SES serves a protective role for cognition (Stern & Konno, 2009).

## Material and methods

Cross-sectional data from the Human Connectome Project (HCP) were used to investigate whether stress, SES, and cognition operated through different models of shared pathways in a tri-ethnic sample of young adults. HCP, a continuing research project started in 2009 and led by Washington University, University of Minnesota, and Oxford University, collected a variety of measures to better understand the human mind (Van Essen et al., 2013). The goals of HCP are many, including to systematically map human brain circuits via multiple neuroimaging modalities, to link such circuits to genes, behaviors, and cognition, and to assess the heritability of neural circuits. Further information on the study overall can be found at https://www.humanconnectome.org/.

### Participants

The original release of data, collected over a three-year period from Washington University, St. Louis, consisted of 1200 subjects between the ages of 22 and 35 years. One goal of the procedure was to be as consistent and yet comprehensive as possible for each participant while not overburdening them. Across two days, participants underwent magnetic resonance imaging and several hours of behavioral assessments (Barch et al., 2013). The assessments included self-report questionnaires to provide information on sociodemographics, personality, emotion, physical and mental functioning, and a battery of cognitive assessments from the National Institutes of Health (NIH) Toolbox (Weintraub et al., 2013). After excluding participants who did not include each of the measures of interest, our sample comprised 970 young adult participants (*mean age* = 29.03, SD = 3.59; 529 females). See Table 1 for sociodemographic information as a function of ethnoracial category.

**Table 1:** Sociodemographic information as a function of ethnoracial category

	Non-Hispanic White Americans	Black Americans	Hispanic Americans
	*M* (SD) or N (%)	M (SD) or N (%)	*M* (SD) or *N* (%)
N	744 (76.70%)	157 (16.19%)	69 (7.11%)
Age	29.16 (3.52)	29.00 (3.61)	27.65 (4.02)
Age range	22–36	22–36	22–36
Sex (% female)	401 (53.90%)	97 (61.78%)	31 (44.93%)
Education	15.13 (1.69)	13.90 (2.10)	14.88 (1.67)
Education range	11–17	11–17	11–17
Income	5.45 (2.00)	3.76 (2.11)	4.93 (2.16)
Income range	1–8	1–8	1–8

### Measures

#### Perceived Stress Scale

To evaluate personal perceptions of stress level, participants completed the PSS (Cohen et al., 1994), a 10-item self-report questionnaire, on an iPad. Questions were answered on a five-point frequency scale (0 = never and 4 =very often). The PSS measures the extent to which an individual feels or perceives situations in their life during the past month as personally stressful. Cohen et al. (1994) reported excellent internal consistency of 0.84 and test–retest reliability of 0.85.

#### Socioeconomic status

Participants’ SES was operationalized as level of education and total household income, which were self-reported through the Semi-Structured Assessment for the Genetics of Alcoholism (SSAGA; Bucholz et al., 1994). For education level, participants reported whether they had less than 11 years of education (< 11), 11, 12, 13, 14, 15, 16, or 17 or more years of education. To measure the level of income, the participants selected the grouping within which their previous yearly total household income fell, coded as < $10,000 = 1, 10 K-19,999 = 2, 20 K-29,999 = 3, 30 K-39,999 = 4, 40 K-49,999 = 5, 50 K-74,999 = 6, 75 K-99,999 = 7, and >  = 100,000 = 8.

### Cognitive assessment

Measures were selected based on previous evidence that ethnoracial differences existed on domains included in the dataset. The assessments were from the NIH Toolbox (Weintraub et al., 2013), and data were provided through the HCP dataset. Except for the *N*-back task, all cognitive assessments were administered on an iPad. Assessments included in all preliminary analyses are listed below.

#### Pattern comparison processing speed test

To evaluate how quickly an individual can process information, participants completed the Pattern Comparison Processing Speed Test (Carlozzi et al., 2014). In this task, participants reported whether two simple images were identical or not as quickly and as accurately as possible. The Pattern Comparison Processing Speed Test has been found to show acceptable test–retest reliability (ranging from 0.46 to 0.74), convergent validity, and discriminant validity (Carlozzi et al., 2014).

#### Flanker Inhibitory Control and Attention Test

In the Flanker Inhibitory Control and Attention Test (Zelazo et al., 2014), participants were shown rows of arrows that varied in number and then indicated which direction the center arrow was pointing by clicking a right or left button. This task is used to measure executive functioning via selective attention and inhibitory control. Previous research has reported good test–retest reliability for the Flanker task, with an interclass correlation of 0.85 (Zelazo et al., 2014).

#### Penn Word Memory Test

The Penn Word Memory Test (Gur et al., 2001, 2010) tapped participants’ verbal episodic memory performance. In this task, participants were presented 20 words and instructed to remember them for a memory test later. After a 20-min delay, participants were presented 40 words—20 presented previously and 20 new words. The participants indicated whether they believed they had seen the word previously by selecting either “definitely yes,” “probably yes,” “probably no,” or “definitely no” responses. Previous research has found the Penn Word Memory Test to have good reliability with an internal consistency of 0.83 (Gur et al., 2001).

#### Picture Sequence Memory Test

The Picture Sequence Memory Test (Dikmen et al., 2014) was used to measure participants’ visual episodic memory, a memory process that involves the acquisition, storage, and retrieval of new visual information. Participants were presented a series of visual pictures of objects and activities in a set order and then were asked to recall the pictures in the correct order. Dikmen et al. (2014) found that the Picture Sequence Memory Test correlated with Rey’s Auditory Verbal Learning test (*r* = 0.64), Brief Visuospatial Memory Test-Revised (*r* = 0.72), and a two-week retest of the Picture Sequence Memory Test performance (*r* = 0.84), demonstrating good construct validity and test–retest reliability.

#### List Sorting Working Memory Test

The List Sorting Working Memory Test (Tulsky et al., 2014) measured working memory capacity, or the ability to selectively maintain and manipulate goal-relevant information without distraction. Participants had a list of objects presented visually and auditorily to them and then verbally repeated all of the objects back to the examiner in order of size—smallest to largest. The List Sorting Working Memory Test evaluates the ability to briefly store information via working memory until storage capacity is reached. Previous research has reported acceptable test–retest reliability for the List Sorting Working Memory Test, with an interclass correlation of 0.77 (Tulsky et al., 2014).

#### N-back task

The *N*-back is a continuous performance task used to evaluate working memory updating—preserving accurate representations of information. In this test, participants were shown a sequence of pictures of tools or faces. For half of the test, participants were asked to indicate if the current picture matched an image presented two pictures prior (2-back). For the other half of the test, participants performed a working memory comparison when asked to indicate if a current picture matched its previous picture (0-back). Unlike the other assessments, this task was performed while the participants were in an fMRI scanner (Van Essen et al., 2013). The *N*-back has been found to show acceptable test–retest reliability ranging from 0.69 to 0.86 (Hockey & Geffen, 2004).

#### Dimensional Change Card Sorting task

The Dimensional Change Card Sort (Frye et al., 1995) captured executive functioning and cognitive flexibility, specifically, switching between tasks quickly. Participants were shown two target cards (e.g., one of a white boat, one of a gold ball) and were asked to match a third card (e.g., gold ball) to one of the target cards based on shape or color. The instruction of matching to shape or to color varied throughout the assessment. Previous research has reported good test–retest reliability for the Dimensional Change Card Sort task, with interclass correlation ranging from 0.90 to 0.94 (Beck et al., 2011).

### Statistical analyses

Data were analyzed using R (Team, 2014). Differences in perceived stress, SES, and each cognitive task as a function of ethnoracial category were assessed using a one-way analysis of variance (ANOVA) with follow-up pairwise comparisons correcting for multiple comparisons using the Bonferroni method. Correlational analyses were first conducted between cognitive performance and our measures of interest, perceived stress and SES, across the whole sample. These correlations were used to confirm that we met criteria for mediation (Baron & Kenny, 1986). Only significant correlations with the task measures were included in subsequent mediation analyses. Mediation and moderated mediation effects were tested using the PROCESS modeling tool in R (Hayes, 2017). For each cognitive task, three mediation analyses were conducted to understand the intertwined relationships among ethnoracial category, stress, SES, and cognition: a parallel mediation model that assumed independent impacts of stress and SES on cognition, a sequential mediation model that assumed that stress operates through SES, and a moderated mediation model that tested whether SES interacted with stress. All mediation analyses controlled for sex (*χ*(2)^2^ = 6.02, *p* = 0.049) and age (*F*(2,967) = 5.63, *p* = 0.004) due to group differences in these variables. Indirect effects are reported as standardized beta coefficients. Outliers were determined through the convergence of multiple measures, including Cook’s distance, leverage values, and Mahalanobis distance, calculated separately for each of the cognitive tasks (6 outliers for picture sequence, 10 for list sorting, 7 for N-back, and 6 for card sorting).

## Results

### Sample characteristics

**Fig. 1:**
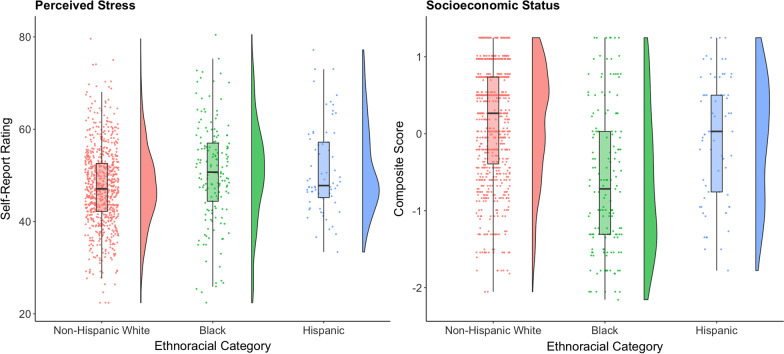
Raincloud plots are presented illustrating ethnoracial category differences in perceived stress (left) and socioeconomic status (right) for Non-Hispanic White Americans (red), Black American (green), and Hispanic Americans (blue)

Across the whole sample, income and education were positively correlated, *r*(964) = 0.36, *p* < 0.001, so a composite SES variable was created by Z-scoring and averaging the two values. As seen in Fig. 1, ethnoracial minorities had greater levels of perceived stress than non-Hispanic White Americans (*p*s < 0.001), but perceived stress did not differ between the two minority groups (*p* = 1.00, after Bonferroni corrections). Non-Hispanic White Americans and Hispanic Americans reported a higher SES than Black Americans (*p* < 0.001), but non-Hispanic White Americans did not differ from Hispanic Americans (*p* = 0.14).

**Fig. 2:**
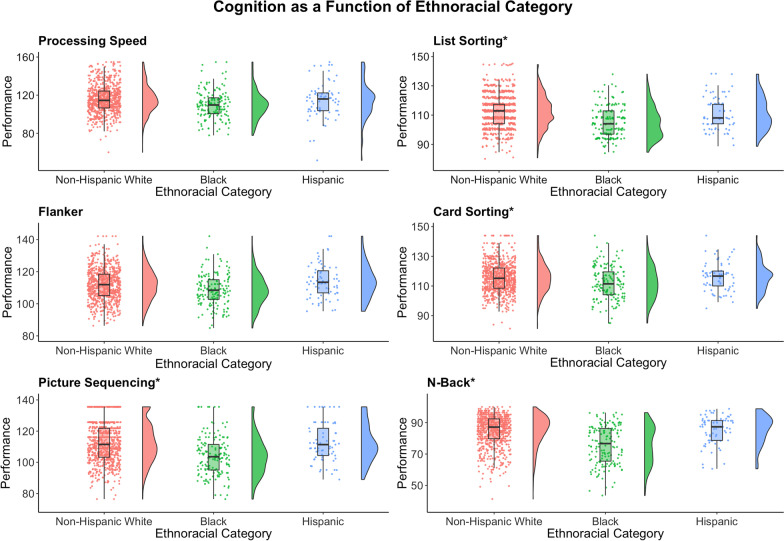
Raincloud plots are presented illustrating ethnoracial category differences in cognition for Non-Hispanic White Americans (red), Black American (green), and Hispanic Americans (blue). The asterisks denote which cognitive tasks also were associated with perceived stress and included in the subsequent mediation analyses. Note that the Penn Word Test did not differ by ethnoracial category, likely due to ceiling effects and so is not included. For completeness, no outliers were removed for these comparisons

For the cognitive tasks, three patterns emerged: 1) non-Hispanic White Americans performed better than Black Americans with Hispanic Americans in between, 2) non-Hispanic White Americans and Hispanic Americans performed better than Black Americans, and 3) no differences between the three groups (see Fig. 2). For processing speed, non-Hispanic White Americans had greater processing speed than Black Americans (*p* < 0.001), but Hispanic Americans fell in the middle and did not significantly differ from either group (*p*s > 0.13). For all other domains except the Penn Word Memory Test, non-Hispanic White Americans did not differ from Hispanic Americans, but both groups performed better than Black Americans (all *p*s < 0.05). For the Penn Word Memory Test, none of the groups differed from each other (all *p*s > 0.26), and therefore no further tests were conducted for this task.

### Zero-order Pearson's correlations

Because our primary focus was how perceived stress was associated with ethnoracial differences in cognition, we specifically focused on significant associations between perceived stress and each of the cognitive tasks. As shown in Table 2, perceived stress was associated with all but two cognitive tasks (processing speed and flanker). Thus, these two cognitive tasks were not tested further. Interestingly, SES was significantly associated with all cognitive tasks but exhibited the weakest associations with processing speed and flanker tasks.

**Table 2:** Zero-order and partial Pearson's correlations across the whole sample

	1	2	3	4	5	6	7	8	9
1. Perceived stress	1.00	− .20***	− .06	− .02	− .07*	− .13***	− .13***	− .12***	− .10***
2. Socioeconomic status	− .20***	1.00	.12***	.11***	.17***	.24***	.26***	.33***	.17***
3. Processing speed	− .06	.09**	1.00	.36***	.11***	.18***	.16***	.26***	.42***
4. Flanker	− .02	.07*	.37***	1.00	.07*	.15***	.12***	.19***	.50***
5. Penn Word Memory	− .07*	.17***	.11***	.06	1.00	.19***	.16***	.21***	.15***
6. Picture Sequence Memory	− .12***	.22***	.18***	.14***	.19***	1.00	.35***	.31***	.19***
7. List sorting	− .13***	.24***	.17***	.13***	,14***	.33***	1.00	.38***	.17***
8. N-Back	− .12***	.28***	.27***	.23***	.19***	.29***	.39***	1.00	.30***
9. Card sorting	− .10***	.13***	.43***	.51***	.14***	.18***	.18***	.32***	1.00

^*^p < .05; **p < .01; ***p < .001. The lower triangle represents the zero-order correlations, and the upper triangle represents partial correlations while controlling for sex and age

### Parallel mediation analyses

We first conducted a series of parallel mediation analyses to predict the extent that perceived stress and SES explain the relationship between ethnoracial category and level of cognition for each of the four cognitive tasks that satisfied the initial mediation assumptions: picture sequence, list sort, *N*-back, and the card sort. This parallel mediation model assumes the perceived stress and SES are relatively independent factors that separately contribute to ethnoracial disparities in cognition.

**Fig. 3:**
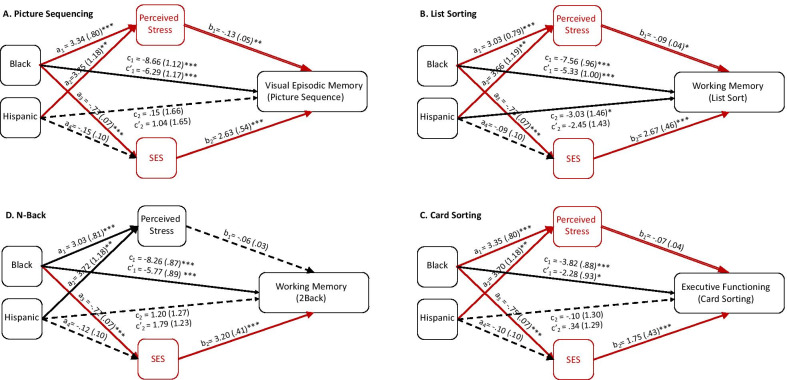
Parallel mediation analyses for picture sequencing (**A**), list sorting (**B**), card sorting (**C**), and the N-Back (**D**). Perceived stress predicted ethnoracial disparities in cognition for the picture sequence task and the list sorting task, but not the other two. SES predicted ethnoracial disparities in cognition for all four cognitive tasks. Unstandardized parameter estimates and standard errors are shown for each pathway. Asterisks denote significant pathways for one ethnoracial minority with a single line and for both ethnoracial minorities with a double line: * = *p* < .05, ** = *p* < .01, *** = *p* < .001. Dashed lines represent non-significant pathways. Red lines and text represent significant mediating pathways

For the picture sequence task (*R*^2^ = 0.10), perceived stress significantly mediated the effect of ethnoracial category on memory performance for both Black Americans (indirect effect =  − 0.034, SE = 0.015, CI [− 0.066, − 0.010]) and Hispanic Americans (indirect effect =  − 0.038, SE = 0.018, CI [− 0.079, − 0.008]). SES also significantly mediated the effect of ethnoracial category on memory performance for Black Americans (indirect effect =  − 0.146, SE = 0.033, CI [− 0.213, − 0.083]) but not for Hispanic Americans (indirect effect =  − 0.030, SE = 0.021, CI [− 0.075, 0.008]) (Fig. 3).

For the list sorting task (*R*^2^ = 0.11), perceived stress significantly mediated the effect of ethnoracial category on list sorting performance for both Black Americans (indirect effect =  − 0.025, SE = 0.013, CI [− 0.056, − 0.003]) and Hispanic Americans (indirect effect =  − 0.030, SE = 0.016, CI [− 0.068, − 0.004]). SES also significantly mediated the effect of ethnoracial category on list sorting performance for Black Americans (indirect effect =  − 0.173, SE = 0.035, CI [− 0.245, − 0.112]), but not for Hispanic Americans (indirect effect =  − 0.021, SE = 0.022, CI [− 0.067, 0.021]).

For the *N*-back task (*R*^2^ = 0.20), perceived stress did not significantly mediate the effect of ethnoracial category on *N-*back performance for either Black Americans (indirect effect =  − 0.017, SE = 0.012, CI [− 0.044, 0.002]) or Hispanic Americans (indirect effect =  − 0.021, SE = 0.015, CI [− 0.053, 0.002]). SES significantly mediated the effect of ethnoracial category on *N*-back performance for Black Americans (indirect effect =  − 0.222, SE = 0.036, CI [− 0.299, − 0.154]), but not for Hispanic Americans (indirect effect =  − 0.037, SE = 0.030, CI [− 0.097, 0.023]).

For the card sorting task (*R*^2^ = 0.07), perceived stress significantly mediated the effect of ethnoracial category on card sorting performance for both Black Americans (indirect effect =  − 0.023, SE = 0.014, CI [− 0.054, − 0.0005]) and Hispanic Americans (indirect effect =  − 0.025, SE = 0.016, CI [− 0.063, − 0.0005). SES also significantly mediated the effect of ethnoracial category on card sorting performance for Black Americans (indirect effect =  − 0.128, SE = 0.034, CI [− 0.198, − 0.064]), but not for Hispanic Americans (indirect effect =  − 0.018, SE = 0.017, CI [− 0.056, 0.015]).

In summary, we found that perceived stress partially mediated the ethnoracial disparities in cognition for three of the four cognitive domains (not the *N*-back task) and for both Black Americans and Hispanic Americans. Independently, SES also partially mediated the ethnoracial disparities in cognition for all the cognitive domains, but only in Black Americans. Notably, the effect sizes for the mediation effects were quite larger for SES than for perceived stress (up to seven times). Overall, these models support the notion that perceived stress and SES contribute to ethnoracial disparities in cognition. However, the direct effects of ethnoracial category on performance were still significant after accounting for both perceived stress and SES for Black Americans (*p*s < 0.007). For Hispanic Americans, the only total effect on cognition that was significant was for the list sorting task and perceived stress fully mediated this effect (Fig. 3﻿).

### Sequential mediation analyses

We next conducted a series of sequential mediation analyses to test whether perceived stress operated through SES to explain the relationship between ethnoracial category and level of cognition for each of the four cognitive tasks.

For the picture sequence task (*R*^2^ = 0.11), the SES-only pathway (indirect effect =  − 0.146, SE = 0.033, CI [− 0.213, − 0.083]) and the SES to perceived stress pathway (indirect effect =  − 0.015, SE = 0.006, CI [− 0.028, − 0.005]) significantly mediated the ethnoracial-cognition disparity for Black Americans. The perceived stress-only pathway did not mediate the ethnoracial-cognition disparity (indirect effect =  − 0.019, SE = 0.012, CI [− 0.045, 0.001]). For Hispanic Americans, the perceived stress-only pathway significantly mediated the ethnoracial-cognition disparity (indirect effect =  − 0.035, SE = 0.017, CI [− 0.073, − 0.007]), but not the SES-only pathway (indirect effect =  − 0.030, SE = 0.021, CI [− 0.075, 0.008]) nor the SES to perceived stress pathway (indirect effect =  − 0.003, SE = 0.002, CI [− 0.009, 0.0008]).

For the list sorting task (*R*^2^ = 0.11), both the SES to perceived stress pathway (indirect effect =  − 0.012, SE = 0.006, CI [− 0.024, − 0.002]) and the SES-only pathway (indirect effect =  − 0.173, SE = 0.035, CI [− 0.245, − 0.112]) significantly mediated the ethnoracial-cognition disparity for Black Americans. The perceived stress-only pathway did not mediate the ethnoracial-cognition disparity (indirect effect =  − 0.013, SE = 0.010, CI [− 0.037, 0.002]). For Hispanic Americans, the perceived stress-only pathway mediated the ethnoracial-cognition disparity (indirect effect =  − 0.028, SE = 0.016, CI [− 0.065, − 0.004]). The SES-only pathway (indirect effect =  − 0.021, SE = 0.022, CI [− 0.067, 0.021]) and the SES to perceived stress pathway (indirect effect =  − 0.001, SE = 0.002, CI [− 0.006, 0.001]) did not significantly mediate the relationship between ethnoracial category and cognition.

For the *N*-back task (*R*^2^ = 0.20), the SES-only pathway mediated the ethnoracial-cognition disparity (indirect effect =  − 0.222, SE = 0.036, CI [− 0.299, − 0.154]), but neither the SES to perceived stress pathway (indirect effect =  − 0.008, SE = 0.005, CI [− 0.019, 0.001]), nor the perceived stress-only pathway (indirect effect =  − 0.009, SE = 0.008, CI [− 0.029, 0.002]) mediated the ethnoracial-cognition disparity for Black Americans. For Hispanic Americans, none of the pathways mediated the ethnoracial-cognition effects (the perceived stress-only pathway: indirect effect =  − 0.019, SE = 0.014, CI [− 0.050, 0.002]; the SES-only pathway: indirect effect =  − 0.037, SE = 0.030, CI [− 0.097, 0.023]; the SES to perceived stress pathway: indirect effect =  − 0.001, SE = 0.002, CI [− 0.005, 0.0009]).

For the card sorting task (*R*^2^ = 0.07), the SES-only pathway (indirect effect =  − 0.128, SE = 0.034, CI [− 0.198, − 0.064]) and the SES to perceived stress pathway (indirect effect =  − 0.010, SE = 0.006, CI [− 0.024, − 0.0002]) significantly mediated the ethnoracial-cognition disparity for Black Americans. The perceived stress-only pathway (indirect effect =  − 0.013, SE = 0.010, CI [− 0.036, 0.001]) did not mediate disparity. For Hispanic Americans, the perceived stress-only pathway mediated the ethnoracial-cognition effects (indirect effect =  − 0.024, SE = 0.016, CI [− 0.060, − 0.0001]). The other two pathways were not significant mediators (the SES-only pathway: indirect effect =  − 0.018, SE = 0.017, CI [− 0.056, 0.015]; the SES to perceived stress pathway: indirect effect =  − 0.001, SE = 0.002, CI [− 0.0059, 0.001]).

**Fig. 4:**
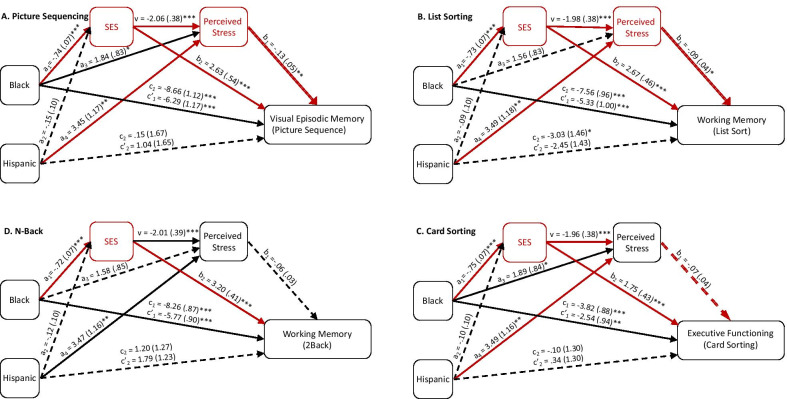
Sequential mediation analyses for picture sequencing (**A**), list sorting (**B**), card sorting (**C**), and the N-back (**D**). Socioeconomic status (SES) predicted perceived stress, which in turn predicted ethnoracial disparities in cognition for the picture sequence task and the working memory task, but not the other two. An independent SES predicted ethnoracial disparities in cognition for all four cognitive tasks. Unstandardized parameter estimates and standard errors are shown for each pathway. Asterisks denote significant pathways for one ethnoracial minority with a single line and for both ethnoracial minorities with a double line: * = *p* < .05, ** = *p* < .01, *** = *p* < .001. Dashed lines represent non-significant pathways. Red lines and text represent significant mediating pathways

In summary, we found that lower SES was associated with greater perceived stress, which contributed an additional pathway mediating ethnoracial differences in cognition for three of the four cognitive domains (not the *N*-back task) for Black Americans. The “SES-only” pathway significantly mediated cognitive disparities in Black Americans for each cognitive domain, but the disparities continued to be significant. For Hispanics, the “perceived stress-only” pathway significantly mediated ethnoracial disparities in cognition for three of the four cognitive domains (not the *N*-back task), but this finding is qualified by the fact that only the list sorting task evidenced significant disparities prior to including these pathways (Fig. 4﻿).

### Moderated mediation analyses

We next conducted a series of moderated mediation analyses to test whether the lack of an independent effect of stress on ethnoracial disparities in cognition might be due to selective effects of stress at lower levels of SES (i.e., SES served as a moderator as well as a mediator). Of critical importance for this final model is the addition of the interaction between SES and perceived stress on cognition.

The perceived stress × SES interaction was not significant for picture sequence (*R*^2^ = 0.11; *b* =  − 0.033, SE = 0.055, *p* = 0.55), list sorting (*R*^2^ = 0.12; *b* =  − 0.039, SE = 0.045, *p* = 0.39), or card sorting (*R*^2^ = 0.07; *b* =  − 0.076, SE = 0.043, *p* = 0.076). For *N*-back (*R*^2^ = 0.20), the perceived stress × SES interaction was significant (*b* = 0.11, SE = 0.042, *p* = 0.012). For Black Americans, the index of moderated mediation was significant (*b* = 0.32, SE = 0.16, CI [0.059, 0.69]) such that perceived stress mediated the ethnoracial disparities in *N*-back performance for those with lower SES (*b* =  − 0.41, SE = 0.20, CI [− 0.87, − 0.09]) but not for those with higher SES (*b* = 0.11, SE = 0.16, CI [− 0.18, 0.45]). For Hispanic Americans, the same pattern was found (moderated mediation index: *b* = 0.39, SE = 0.21, CI [0.063, 0.88]; lower SES: *b* =  − 0.51, SE = 0.26, CI [− 1.11, − 0.10]; higher SES: *b* = 0.13, SE = 0.20, CI [− 0.21, 0.57]). However, the direct effect of ethnoracial category on *N*-back performance was still significant in Black Americans (*p* < 0.001). Consistent with our hypotheses, higher SES appears to partially protect against stress-related declines in cognition, at least for the *N*-back task.

**Fig. 5:**
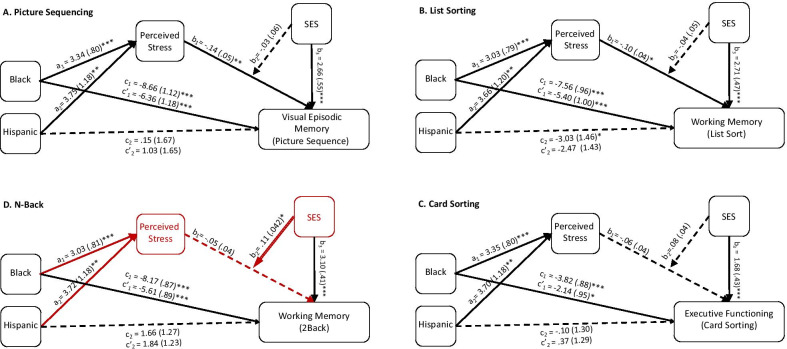
Moderated mediation analyses for picture sequencing (**A**), list sorting (**B**), card sorting (**C**), and the N-back (**D**). Socioeconomic status (SES) moderated the effect of perceived stress on ethnoracial disparities in cognition only for card sorting and the N-back task. Unstandardized parameter estimates and standard errors are shown for each pathway. Asterisks denote significant pathways for one ethnoracial minority with a single line and for both ethnoracial minorities with a double line: * = *p* < .05, ** = *p* < .01, *** = *p* < .001. Dashed lines represent non-significant pathways. Red lines and text represent significant mediating pathways

In summary, we found evidence that the mediating effect of perceived stress on the ethnoracial category–cognition relationship depended on SES for the *N-*back task but not any of the other tasks for both Black Americans and Hispanic Americans. Across the two groups, the direction of the effect was similar: the lower the levels of SES, the more likely stress mediated the ethnoracial disparities in cognition (Fig. 5).

## Discussion

The present study investigated how stress and SES impact ethnoracial differences in cognition among Black Americans and Hispanic Americans relative to non-Hispanic White Americans during young adulthood. Systemic racism and discrimination often experienced by ethnoracial minorities have been proposed as critical adversities that lead to premature weathering of health, perhaps impacting cognition (Brody et al., 2014; Forde et al., 2019). Given that few studies have tested mediating pathways between stress and SES on outcomes such as cognition (Cundiff et al., 2020), we examined three critical pathways in which stress and SES might influence ethnoracial differences in cognition: SES and stress independently (parallel mediation), SES-induced stress (sequential mediation), or through stress but only for lower SES individuals (moderated mediation). Support was found for each of these patterns: independent effects of each, stress through SES, and stress dependent on the level of SES. Although we revealed significant mediation effects for both Black and Hispanic Americans, none of the mediating pathways completely mediated the ethnoracial disparities for Black Americans, and Hispanic Americans in our sample did not show strong evidence of cognitive disparities relative to non-Hispanic White Americans apart from the list sorting task. For this reason, we focus much of the discussion on patterns across the entire sample or differences between Black Americans and non-Hispanic White Americans.

### Two stress pathways in ethnoracial minorities

The sequential mediation analyses proved to be most informative for the episodic memory, working memory capacity, and executive function domains because these analyses allowed the assessment of the relationship between SES and stress as well as the independent influence of each on cognition. Across the whole sample, lower SES was associated with greater stress. Although the independent effect of stress on ethnoracial disparities in cognition was significant in the parallel models, once the SES–stress relationship was included in the model (via sequential mediation), stress alone only exerted an independent effect in Hispanic Americans and was no longer significant for Black Americans. Thus, the effects of stress on cognition were likely due to stress brought about by being in a lower socioeconomic stratum in Black Americans and non-Hispanic White Americans. Compared to non-Hispanic White Americans, Black Americans often face more financial stress, which likely derives from a lower SES (The Pew Charitable Trusts, 2015). Furthermore, financial stress and SES may directly impact physical and mental health through limited access to proper healthcare and a lack of self-care (Dressler et al., 2005). This example and others are only exacerbated by systemic racism that prevents many Black Americans from improving their social standing by moving up in class (e.g., housing segregation, policing; Salter et al., 2018).

As mentioned above, this effect was found for all cognitive tasks except working memory updating. This differential association based on cognitive domain might be due to the greater influence of stress on hippocampal functioning relative to other brain regions (Piccolo et al., 2018). Indeed, the hippocampus is well known for its role in storing and retrieving episodic memories (Burgess et al., 2002), as would be measured by the picture sequencing task. However, research in patient populations and neuroimaging also has shown that the hippocampus is necessary for holding information in short-term memory (Cave & Squire, 1992). Although executive function is canonically associated with the prefrontal cortex, many studies have also shown the involvement of the hippocampus, suggesting that complex tasks like the card sorting task used in the present study rely on many different brain regions (Nyhus & Barceló, 2009). In contrast, working memory updating such as the *N*-Back task frequently does not activate the hippocampus (Blokland et al., 2008) and actively suppresses hippocampal activity as working memory load increases (Stretton et al., 2012). The reliance on frontoparietal brain regions during working memory updating might lead to weaker influences of stress on cognitive processing in these domains. The *N*-Back task also stands out as an outlier because it was the only task given during the fMRI scanning session, which can alter behavioral performance (Gutchess & Park, 2006). Although the differences in tasks remain unclear, the present results nevertheless show that SES can cause stress-related differences in a variety of cognitive domains.

We also found that stress impacted cognition differently depending on the SES of the participants. More specifically, the lower level of SES, the more likely stress mediated the ethnoracial disparities in working memory updating. In contrast to the sequential SES to stress pathway found uniquely in Black Americans, this effect occurred jointly in Black and Hispanic Americans. This stress pathway suggests that some types of stress related to cognition are unique to ethnoracial minorities with a lower SES relative to minorities in a higher SES. Lower SES individuals often occupy jobs that lack independence and personal control (Kraus et al., 2012). The lower material resources that lower SES adults have only further fuels such feelings, which increases one’s perceived stress (Kraus et al., 2009). Additionally, the intersection of lower SES and minority status might give rise to greater stress related to discrimination. Consistent with this idea, many marginalized identities have been related to posttraumatic stress, lower quality of life, and discrimination (Crenshaw, 1989; Seng et al., 2012).

The other side of this moderation effect suggests that ethnoracial minorities with higher SES do not show an association between stress and cognition. Resources of higher-SES in these individuals may afford greater resilience or cognitive reserve that helps protect cognition (Stern & Konno, 2009). For example, more financial resources would allow better quality or longer therapy to cope with stress. Additionally, greater education may also bring knowledge for engaging in better health behaviors that can keep the brain healthy, thereby improving cognition (see also, Cundiff et al., 2020). Higher SES may also afford more opportunities for social support that can buffer against stress (Cohen & McKay, 2020).

### Independent effects of SES on ethnoracial disparities in cognition

Across the whole sample, SES also evidenced an independent association with cognition. Specifically, lower SES was associated with lower levels of cognition, and this finding was significant in each of the cognitive tasks in Black Americans, suggesting a more general (vs. domain specific) effect. For example, SES has been related to cortical thickness across the whole brain in adolescents (Mackey et al., 2015) and to gray matter density in medial prefrontal regions in young adults (Yang et al., 2016; for review see, Merz et al., 2019). This SES-cognition pathway was independent of the pathway related to stress. A partial list of other mechanisms by which SES may contribute to lower cognition includes fewer educational resources starting in childhood, less access to healthy foods, gestational effects that might lead to lower birth weight (and thus lower overall health), exposure to second-hand smoke, or adoption of smoking and other poor health habits (Dressler et al., 2005; Glymour & Manly, 2008; Merz et al., 2019).

Given that many of the above factors disproportionately negatively impact Black Americans, it is perhaps not surprising that these “other” SES effects also partially mediated the cognitive disparities between Black Americans and non-Hispanic White Americans. These SES-related associations with lower cognition may have long-lasting impacts on cognitive levels throughout the adult lifespan. Many studies have documented the lower level of cognition in older Black Americans compared with non-Hispanic White Americans (Early et al., 2013; Gross et al., 2015; Manly et al., 1998; Weuve et al., 2018; Zaheed et al., 2020; Zahodne et al., 2017). The present study suggests that these relationships among older Black Americans likely originate much earlier in life and are not due to differential rates of longitudinal decline between older Black Americans and older non-Hispanic White Americans. For example, SES differences in brain structure and function have been observed even in the first year of life (Merz et al., 2019). At the same time, Black Americans are, on average, more likely to develop Alzheimer’s disease and related dementias compared with non-Hispanic White Americans (Mindt et al., 2013; Perkins et al., 1997). Therefore, the SES-related associations with lower cognition could be related to lower cognitive reserve (also often measured by SES) that is thought to protect older adults from cognitive decline due to dementia (Stern & Konno, 2009).

### Generalizability of results

The sample of participants used in the present study was not nationally representative; instead, participants all resided in Missouri. Although efforts were made to ensure that participants reflected ethnoracial compositions within the US (Van Essen et al., 2013), one cannot conclude that the ethnoracial minorities in the sample generalize to ethnoracial minorities in other parts of the country. Relatedly, no additional information was available in the present dataset to test for heterogeneous effects among Black Americans and Hispanic Americans (cf. McDonough et al., 2021). For example, many cultural practices, beliefs, and health behaviors differ among African-Americans, US-born Caribbean Blacks, and foreign-born Caribbean Blacks (Chatters et al., 2009; Mouzon & McLean, 2017; Rong & Brown, 2001). Thus, although generalizability is questionable, participants within each ethnoracial category had the full range of measured education and income levels, thereby representing people from all socioeconomic strata. This variability was sufficient to replicate correlations between ethnoracial minorities and socioeconomic status, perceived stress, and cognition as found in larger and more nationally representative samples (e.g., Oumohand et al., 2020; Zahodne et al., 2017, 2019).

Another exception to the generalizability of the results was the inconsistent cognitive disparities between Hispanic Americans and non-Hispanic White Americans (e.g., Díaz-Venegas et al., 2019). One major factor worth reiterating is that not all Hispanic Americans are a part of a homogeneous group; therefore, subgroups might not evidence cognitive disparities relative to non-Hispanic White Americans (McDonough et al., 2021). An additional factor explaining this null result is the relatively small sample size. The sample consisted of over twice as many Black Americans as Hispanic Americans. We chose to include the Hispanic Americans in the analysis rather than remove them a) to be inclusive of other ethnoracial minorities and b) to increase our total sample size given that a single model was used to test our relationships rather than separating the mediation models into separate subgroups. Despite the smaller sample size, we did show significant interaction effects between SES and stress for Hispanic Americans and the significant mediating effects of the “stress-only” pathway did show the expected patterns in the list sorting task. We also note that the cognitive measures were collected in person and through reliable tests (via the NIH Toolbox) that potentially can reduce measurement noise compared to larger national datasets in which cognition relies on telephone interviews and less reliable measures (Lachman et al., 2014; Ofstedal et al., 2005).

### Other considerations

Another factor worth considering is that neither SES nor stress completely accounted for the cognitive disparities found between Black Americans and non-Hispanic White Americans. One possibility is that different measures of SES or stress might capture complementary sources of variance in cognitive disparities. For example, objective measures of chronic stress such as event checklists or cortisol derived from hair samples might provide a more long-term perspective of how stress and SES interface to predict cognition (Staufenbiel et al., 2013; Turner & Wheaton, 1995). Another possibility is that although both stress and SES are important sources of variance in cognition, the longstanding and widespread impact of systemic racism on Black Americans cannot be boiled down to these two factors alone. Indeed, the NIH Health Disparities Research Framework (Hill et al., 2015) outlined 62 factors across 12 categories that might begin explaining ethnoracial disparities. Although this framework was intended to pertain to health generally (as was the weathering hypothesis), we view cognition as a type of health to which this framework can readily be applied (cf. McDonough, 2017; McDonough et al., 2021). The present study provides continued evidence for lifelong effects of cognitive disparities that are shaped by factors largely outside of one’s control.

### Clinical implications

The present findings also have implications for clinical settings. In cognitive or neuropsychological assessment, norms are essential in deciphering where an individual stands among the population. Our study showed an ethnoracial disparity in cognitive functioning, which speaks to the need for ethnoracially based norms, or more importantly, clinical practices that factor ethnoracial disparities into clinical decision-making. For example, Black Americans can be falsely diagnosed with cognitive impairment if the cognitive test and the clinician do not take ethnoracial disparities into consideration; this could partly explain why Black Americans have a higher prevalence of dementia than White Americans (Hayward et al., 2021). Furthermore, we used data from young adults for the current study, and the disparity was still noticeable, indicating the role of early developmental factors in late-life cognitive performance. Thus, when diagnosing cognitive disorders more commonly seen in young adults (e.g., traumatic brain injury, autism), ethnoracial disparity should also be factored into clinical decision-making.

## Conclusion

While much research has revealed cognitive disparities in some ethnoracial minorities in childhood and in advanced age, fewer studies have investigated whether such disparities might be minimized in young adulthood when the brain has reached full maturity and optimal cognitive performance. The present study revealed that, at least in this sample of Black Americans, cognitive disparities could be quite prominent in this age range. Furthermore, we found two different pathways that partially explain such disparities in Black Americans: an SES-dependent stress pathway and an independent SES pathway. Separately, stress appeared to be a factor associated with poorer cognition in Hispanic Americans independent from SES. Even though both SES and stress have been strongly influenced by a history of systemic racism that increases barriers to education and work opportunities, many other factors exist that have an additional influence to maximize cognitive outcomes among ethnoracial minorities. These lifelong negative influences might put some ethnoracial minorities at greater risk for cognitive impairments such as late-life dementia.

## Data Availability

The datasets supporting the conclusions of this article can be requested from humanconnectome.org.

## Abbreviations

ANOVA: Analysis of variance
fMRI: Functional magnetic resonance imaging
HCP: Human Connectome Project
HPA: Hypothalamic–pituitary–adrenal
NIH: National Institutes of Health
PSS: Perceived Stress Scale
SES: Socioeconomic status
